# Investigating TSPO levels in occupation-related posttraumatic stress disorder

**DOI:** 10.1038/s41598-023-31327-y

**Published:** 2023-03-27

**Authors:** Sarah E. Watling, Talwinder Gill, Erin V. Gaudette, J. Don Richardson, Tina McCluskey, Junchao Tong, Jeffrey H. Meyer, Jerry Warsh, Rakesh Jetly, Michael G. Hutchison, Shawn G. Rhind, Sylvain Houle, Stephen J. Kish, Isabelle Boileau

**Affiliations:** 1 Institute of Medical Sciences, University of Toronto, Toronto, ON Canada; 2 Brain Health Imaging Centre, Centre for Addiction and Mental Health, Toronto, ON Canada; 3 Campbell Mental Health Research Institute, Centre for Addiction and Mental Health, Toronto, ON Canada; 4 Department of Psychiatry, University of Toronto, Toronto, ON Canada; 5 Department of Pharmacology and Toxicology, University of Toronto, Toronto, ON Canada; 6 The MacDonald Franklin OSI Research Centre, Lawson Health Research Institute, London, ON Canada; 7 Department of Psychiatry, University of Western Ontario, London, ON Canada; 8 Department of Psychiatry and Behavioural Neurosciences, McMaster University, Hamilton, ON Canada; 9 St Joseph’s, London OSI, Parkwood Institute, St. Joseph’s Health Care, London, ON Canada; 10 Directorate of Mental Health, Canadian Forces Health Services, Ottawa, ON Canada; 11 Department of Psychiatry, Faculty of Medicine, University of Ottawa, Ottawa, ON Canada; 12 Department of Psychiatry, Faculty of Medicine, Dalhousie University, Halifax, NS Canada; 13 Faculty of Kinesiology and Physical Education, University of Toronto, Toronto, ON Canada; 14 David L. MacIntosh Sport Medicine Clinic, Faculty of Kinesiology and Physical Education, University of Toronto, Toronto, ON Canada; 15 Keenan Research Centre for Biomedical Science of St. Michael’s Hospital, Toronto, ON Canada; 16 Defence Research and Development Canada, Toronto Research Centre, Toronto, ON Canada

**Keywords:** Neuroscience, Molecular neuroscience, Neuroimmunology

## Abstract

Microglia are immune brain cells implicated in stress-related mental illnesses including posttraumatic stress disorder (PTSD). Their role in the pathophysiology of PTSD, and on neurobiological systems that regulate stress, is not completely understood. We tested the hypothesis that microglia activation, in fronto-limbic brain regions involved in PTSD, would be elevated in participants with occupation-related PTSD. We also explored the relationship between cortisol and microglia activation. Twenty participants with PTSD and 23 healthy controls (HC) completed positron emission tomography (PET) scanning of the 18-kDa translocator protein (TSPO), a putative biomarker of microglia activation using the probe [^18^F]FEPPA, and blood samples for measurement of cortisol. [^18^F]FEPPA V_T_ was non-significantly elevated (6.5–30%) in fronto-limbic regions in PTSD participants. [^18^F]FEPPA V_T_ was significantly higher in PTSD participants reporting frequent cannabis use compared to PTSD non-users (44%, p = 0.047). Male participants with PTSD (21%, p = 0.094) and a history of early childhood trauma (33%, p = 0.116) had non-significantly higher [^18^F]FEPPA V_T_. Average fronto-limbic [^18^F]FEPPA V_T_ was positively related to cortisol (r = 0.530, p = 0.028) in the PTSD group only. Although we did not find a significant abnormality in TSPO binding in PTSD, findings suggest microglial activation might have occurred in a subgroup who reported frequent cannabis use. The relationship between cortisol and TSPO binding suggests a potential link between hypothalamic–pituitary–adrenal-axis dysregulation and central immune response to trauma which warrants further study.

## Introduction

Posttraumatic stress disorder (PTSD), a common disorder that can develop after exposure to a traumatic event, affects approximately 9% of Canadians^1^ and up to 30% of adults in at-risk cohorts (e.g., first responders and military members)^2^; a rate which increased substantially during the coronavirus (COVID-19) pandemic^3^. The clinical features of PTSD (e.g., nightmares, negative mood, hyperarousal)^4^, and lack of effective pharmacological treatment^5^ make this psychiatric disorder extremely debilitating.

Microglia, the brain’s immune cells^6^, have been implicated in psychiatric disorders^7^. These central immune cells are key mediators of neuroinflammation^8^; for example, in response to injuries or toxins, microglia change from a resting to an activated state in which they carry out their immune functions. Moreover, evidence suggests that intense psychological stress elicits microglia activation in brain regions associated with threat appraisal and emotional responses, and alters their phenotypic and functional properties via sterile neuroinflammatory pathways^9,10^. Despite some conflicting findings^11–14^, preclinical models of PTSD, for the most part, suggest that acute and chronic stress promote structural and proteomic changes in microglia with the release of proinflammatory mediators [e.g., interleukin (IL)-1β, IL-6 and tumor necrosis factor (TNF)-α)]^15^ and cytotoxic factors^16^. Specifically, both increased levels of ionized calcium binding protein (IBA)-1, a protein specific to microglia that upregulates upon activation^17^, and proliferation of morphologically hypertrophic microglia^18–21^ have been reported in key brain areas [i.e., hippocampus, amygdala, and prefrontal cortex (PFC)] in rodent models of PTSD. In line with these findings, post-mortem research has reported decreased secretion of the pro-inflammatory cytokine, IL-1α, by activated microglia in the dorsolateral prefrontal cortex (DLPFC)^22^, and decreased expression of microglia related genes in females with PTSD^23^. Interestingly, in preclinical models of PTSD^20,24^ and in one case study in PTSD^25^, exposure to minocycline, an antibiotic that can inhibit microglia activation, alleviates anxiety-related behavior and PTSD symptoms.

Positron emission tomography (PET) imaging, targeting the 18-kDa translocator protein (TSPO), enables the investigation of microglia in living participants^26^. TSPO is a mitochondrial membrane protein distributed throughout the central nervous system, primarily on microglia cells (but also in astrocytes and endothelial cells). Expression of TSPO increases during microglia activation^27^, thus TSPO has been used as a biomarker of microglia activation. To date, findings of TSPO binding in PTSD are conflicting. Bhatt et al.^23^ reported decreased TSPO binding in participants diagnosed with PTSD, while Deri et al.^28^ reported a positive relationship between TSPO binding and PTSD symptom severity in World Trade Centre responders (with and without a diagnosis of PTSD). Similarly, Seo et al.^29^ reported higher TSPO binding in fibromyalgia participants endorsing more PTSD symptoms. These conflicting reports suggest that more research is needed to understand the role of microglia in PTSD, as well as the mechanisms by which microglial dysfunction might occur. In this regard, glucocorticoids (GCs), downstream effectors of stress responses through the hypothalamic pituitary adrenal (HPA) axis, have been implicated in the pathophysiology of PTSD and are known to act on microglia in both pro- and anti-inflammatory capacities^30^. The putative effects of GCs on brain immune responses in PTSD are of significant interest. Currently, there are very few studies investigating the association between peripheral cortisol (primary GC) and microglia in living humans. The aim of the current study was to test whether higher TSPO binding in the fronto-limbic neurocircuitry, implicated in stress and emotional regulation, occurs in occupation-related PTSD by employing the fluorine F 18–labeled radiotracer [^18^F]FEPPA (*N*-(2-(2-fluoroethoxy)benzyl)-*N*-(4-phenoxypyridin-3-yl)acetamide). We also investigated whether TSPO binding is related to clinical symptoms and serum cortisol concentrations.

## Results

### Participants

Twenty participants with a diagnosis of PTSD and 23 Healthy Controls (HC) met criteria and completed [^18^F]FEPPA PET scans with arterial blood sampling and an MRI scan. Three PTSD PET scans and one HC PET scan were excluded due to poor fit of the time activity curve (TAC) in the kinetic modelling: thus, 17 PTSD [^18^F]FEPPA PET scans and 22 HC [^18^F]FEPPA PET scans were included in the analysis. All participant demographics and scan parameters can be found in Table 1 and PTSD clinical characteristics in Table 2. PTSD participants (mean age 44 years) were older than HC (mean age 34.6 years) but were matched on sex, race, body mass index (BMI), TSPO genotype, years of education and cigarette smoking. While there was a similar number of regular cannabis users in each group (PTSD: n = 6, HC: n = 7), PTSD cannabis users reported using more frequently and in higher doses (Table 1). Additionally, 7 PTSD participants tested positive for tetrahydrocannabinol (THC) on their PET scan day compared to 4 HC participants. PTSD participants reported significantly higher symptoms of anxiety and depression (p < 0.001) compared to HC participants as measured by the Generalized Anxiety Disorder-7 (GAD-7^31^) questionnaire, Patient Health Questionnaire-9 (PHQ-9^32^), and Beck Depression Inventory (BDI^33^).

**Table 1:** Participant demographics: PTSD and HC.

	PTSD (n = 17)	HC (n = 22)	p value
Age, years	44 ± 10.1	34.6 ± 13.9	0.01^A^
Sex, male n (%)	8 (47)	11 (47)	0.829^C^
NIH race, Caucasian n (%)	13 (76)	12 (52)	0.591^C^
BMI (kg/m^2^)	26.5 ± 4	24.8 ± 4.1	0.195^A^
TSPO genotype, HAB n (%)	8 (47)	12 (54)	0.678^C^
Years of education	15.6 ± 2.9	15.5 ± 2	0.912^A^
Cigarette smokers, n (%)	3 (17) (4 former smokers)	1 (4) (2 former smokers)	0.651^C^
Positive THC on PET day, n (%)	7 (41)	4 (23)	0.123^C^
Current Cannabis use, n (%)	6 (35) [3 daily]	7 (30)	0.914^C^
Cannabis (g/week)	17.9 ± 30.2	2.3 ± 2.1	0.377^A^
Alcohol (drinks/week)	2.71 ± 2.4	2.69 ± 1.9	0.959^A^
*Questionnaires*			
BDI, median (range)	21.7 (4–39)^**+**^	2.4 (1–4.5)^**+**^	< 0.001^A^
GAD-7, median (range)	9.3 (0–21)*	0 (0–2.5)*	< 0.001^A^
PHQ-9, median (range)	12.3(1–27)*	3 (0.5–3)*	< 0.001^A^
*Scan parameters, mean (range)*			
Amount injected (mCi)	4.9 (4.49–5.29)	5.1 (4.63–6.62)	0.02^B^
Specific activity at time of injection (mCi/μmol)	4575 (1053–9395)	3188 (1047–7825)	0.134^B^
Mass injected (μg)	0.91 (0.23–2.53)	0.58 (0.13–1.7)	0.06^B^

Values are mean ± SD unless otherwise indicated.

Evaluated by T-Test^A^ , Mann–Whitney U Test^B^, Chi Square Test^C^.

PTSD: n = 10* & n = 20 + .

HC: n = 15* & n = 14 + .

*BMI* Body mass index, *HAB* high affinity binder, *BDI* Beck Depression Inventory, *GAD-7* General Anxiety Disorder-7, *g* grams, *NIH* National Institute of Health, *PHQ-9* Patient Health Questionnaire-9, *THC* tetrahydrocannabinol, *TSPO* translocator protein.

**Table 2:** PTSD characteristics.

	PTSD (n = 17)
PSS, median (IQR)	32 (15–44)
PCL, median (IQR)	59 (37–68)
Duration of PTSD (years)	4.9 ± 3.7
Age of onset (years)	30 ± 11.7
Current MDD, n (%)	5 (29)
History of mTBI, n (%)	8 (47)
*Medication use, n (%*)	15 (88)
Cannabis	5 (29)
SSRI	10 (61)
SARI	2 (11)
SNRI or NDRI	2 (11)
Atypical antipsychotics	2 (11)
Benzodiazepines	2 (11)
PDE5 inhibitor	1 (5)
Alpha blocker	1 (5)
*Occupation, n (%*)	
Correctional officer	1 (5)
Nurse	2 (11)
Paramedic	2 (11)
Police officer	2 (11)
Military	10 (58)
*Lifetime trauma exposure, n (%)*	
Natural disaster	9 (53)
Early childhood trauma	9 (53)
Physical violence	15 (88)
Sexual violence	3 (18)
Accident	13 (76)
War zone	10 (58)

Values are mean ± SD unless otherwise indicated.

*IQR* Interquartile range, MDD major depressive disorder, *mTBI* mild traumatic brain injury, *NDRI* norepinephrine-dopamine reuptake inhibitors, *PDE5* phosphodiesterase type 5, *PSS* PTSD Symptom Scale, *PCL* PTSD checklist, *SARI* serotonin antagonist and reuptake inhibitors, *SNRI* serotonin-norepinephrine reuptake inhibitor, *SSRI* selective serotonin reuptake inhibitor.

PTSD participants in the current study were highly symptomatic with PTSD Checklist (PCL) scores (median 59) indicating ‘Severe’ PTSD (PCL score > 45 is classified as severe). PTSD participants had been coping with the psychiatric disorder for approximately 5 years, with an average onset age of 30 years. Five participants (29%) had current co-morbid major depressive disorder (MDD) and 8 participants (47%) reported a history of mild traumatic brain injury (mTBI). The majority of participants with PTSD (88%) were taking medication, the most common medication being Selective Serotonin Reuptake Inhibitors (SSRIs, 61%), followed by cannabis (29%).

### Model diagnostics

**Figure 1:**
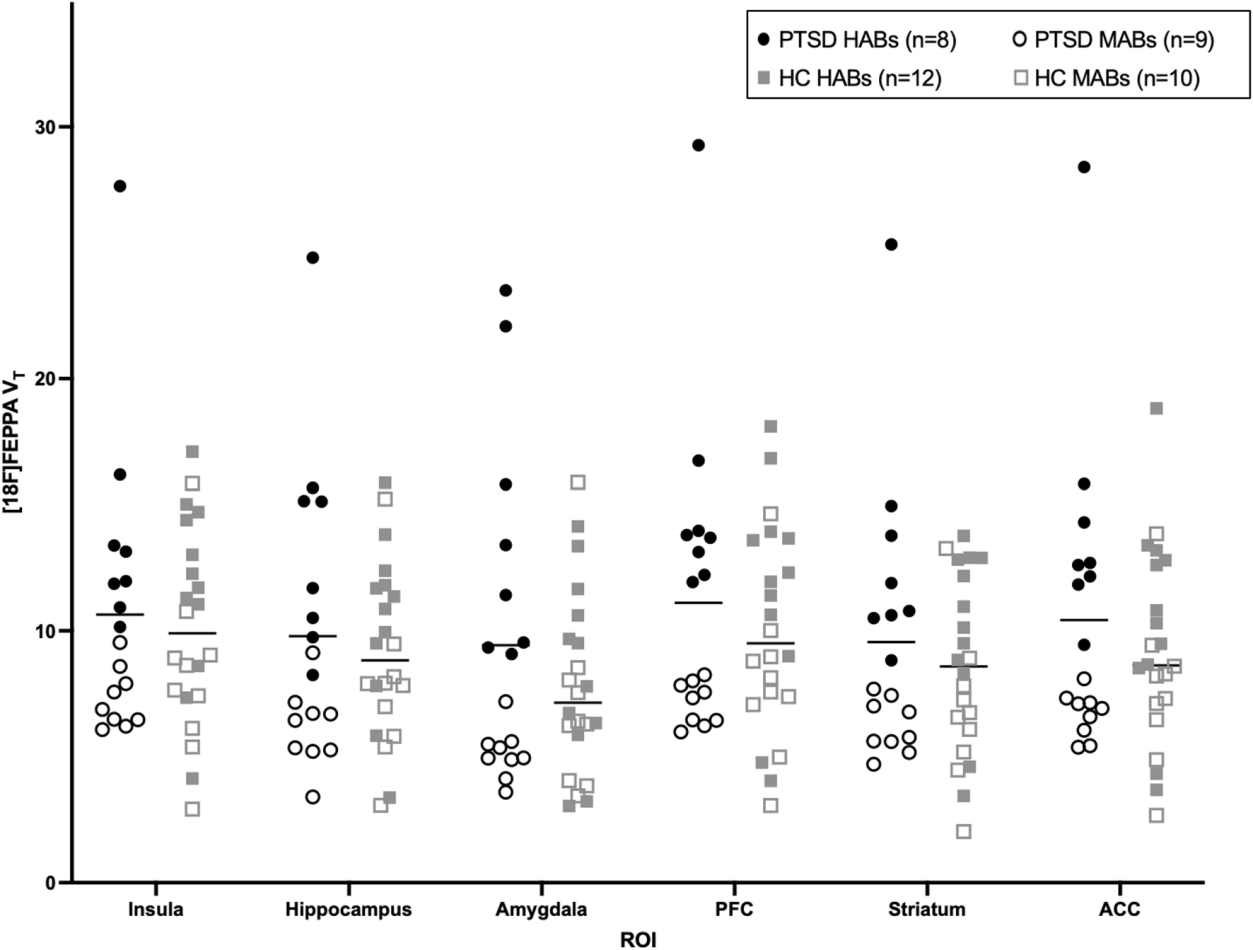
Group differences in [^18^F]FEPPA V_T_. [^18^F]FEPPA V_T_ scatterplot between PTSD (n = 8 HABs [solid]/9 MABs [outline]) and Healthy Controls (HC) (n = 12 HABs [solid]/10 MABs [outline]). RM ANCOVA controlling for genotype and age revealed no main effect of group (F_(1,35)_ = 1.123, p = 0.297) and a marginal ROI × Group interaction (F_(2.645,92.571)_ = 1.954, p = 0.134). Pairwise comparisons: Insula (6.5% difference, p = 0.596); Hippocampus (12% difference, p = 0.279); Amygdala (30% difference, p = 0.107); PFC (16% difference, p = 0.406); Striatum (22% difference, p = 0.328); and ACC (15% difference, p = 0.356).

[^18^F]FEPPA V_T_ data in the 6 regions of interest (ROIs) of interest was not normally distributed (p < 0.05). Upon visual inspection of the data (Fig. 1), there was one data value that appeared to be an outlier in all ROIs. Z score > 3 and Cooks Distance > 0.15 confirmed this data point was an outlier and an influential value in our RM-ANCOVA. Since exclusion of this data point did not change our findings and as our sample size was small, we continued with our primary analysis including all data points. We subsequently conducted a sensitivity analysis where the influential data point was assigned the second greatest [^18^F]FEPPA V_T_ value and therefore no longer influencing the model.

### Is TSPO binding higher in PTSD?

A RM-ANCOVA controlling for TSPO genotype and age revealed no main effect of group (F_(1,35)_ = 1.123, p = 0.297) and a marginal ROI × Group interaction (F_(2.645, 92.571)_ = 1.954, p = 0.134). While age was not a significant covariate in the model (F_(1,35)_ = 0.665, p = 0.420), our PTSD group was older than our HC group (p = 0.01) and inclusion of age improved the model [inclusion of sex did not improve the model (F_(1,35)_ = 0.696 p = 0.410)]. Additionally, visual inspection of the data (Fig. 1) revealed overlap in [^18^F]FEPPA V_T_ values between PTSD and HC groups. Genotype and age adjusted [^18^F]FEPPA V_T_ means were between 6.5 and 30% higher in fronto-limbic ROIs (insula, hippocampus, amygdala, PFC, striatum, and ACC) in PTSD participants. The strongest pairwise comparison was in the amygdala, where age and genotype adjusted TSPO binding was 30% higher in PTSD (p = 0.107). As expected, the TSPO genotype explained a significant amount of variance in [^18^F]FEPPA V_T_ (p < 0.0001), with HABS having significantly higher [^18^F]FEPPA binding than MABs (Fig. 1). There was no effect of amount injected (mCi) or mass on [^18^F]FEPPA V_T_.

Sensitivity analysis revealed no main effect of group (F_(1,35)_ = 0.472, p = 0.496) and a significant ROI × Group interaction (F_(2.563,89.704_ = 3.957, p = 0.015) (see Supplementary Fig. S1). The strongest pairwise comparison was in the amygdala (p = 0.114) where PTSD participants had 29% higher [^18^F]FEPPA V_T_ compared to HC.

### Do SSRIs, cannabis use, current MDD, history of mTBI, or early childhood trauma affect TSPO binding in PTSD participants?

**Figure 2:**
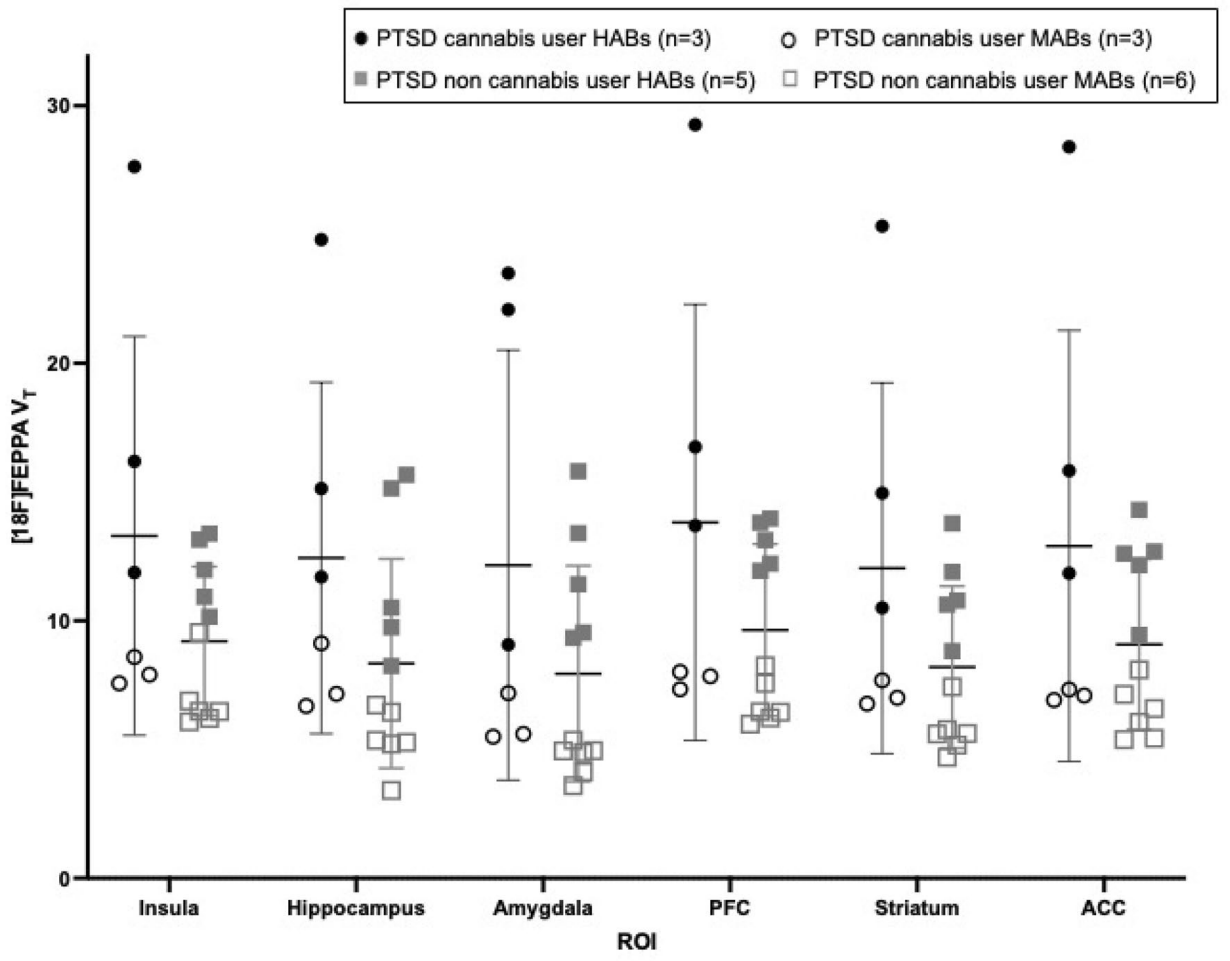
[^18^F]FEPPA V_T_ and cannabis use. [^18^F]FEPPA V_T_ between PTSD participants who regularly consume cannabis (n = 3 HABs [solid]/n = 3 MABs [outline]) and PTSD participants who are non-cannabis users (n = 5 HABs [solid]/n = 6 MABs [outline]). PTSD cannabis users have 41.6% higher TSPO binding compared to PTSD participants who do not use cannabis (F_(1,14)_ = 4.759, p = 0.047).

A RM-ANCOVA in PTSD participants revealed no effect of history of mTBI (mTBI, n = 8 vs no mTBI, n = 9: F_(1,14)_ = 0.923, p = 0.353) or current MDD (MDD, n = 5 vs no MDD, n = 12: (F_(1,14)_ = 0.007, p = 0.935) on [^18^F]FEPPA V_T_. In contrast, SSRI use (SSRI use, n = 10 vs no SSRI use, n = 7) was marginally significant (F_(1,14)_ = 3.890, p = 0.069) such that participants with PTSD on SSRIs had 27% lower [^18^F]FEPPA V_T_ compared to participants not on SSRIs and showed no difference from HC (p = 0.837). A RM-ANCOVA between PTSD with regular cannabis use (n = 6) and PTSD without cannabis use (n = 11) (see Supplementary Table S1) revealed a significant main effect of group where regular cannabis users had 41.6% higher [^18^F]FEPPA V_T_ compared to PTSD participants who do not use cannabis (F_(1,14)_ = 4.759, p = 0.047) and 38% marginally higher [^18^F]FEPPA V_T_ compared to HC (F_(1,24)_ = 3.666, p = 0.068) (Fig. 2). A RM-ANCOVA between PTSD participants with (n = 9) and without (n = 8) a history of early childhood trauma (ECT) revealed a marginal main effect of group (F_(1,14)_ = 2.812, p = 0.116) where PTSD + ECT had 33% higher [^18^F]FEPPA V_T_ compared to those without. The strongest main group effect was observed in the ACC (% difference: 38%, p = 0.091). Sensitivity analysis controlling for the outlier only changed two of the findings reported above: the group difference between PTSD participants currently taking and not taking SSRIs became significant (p = 0.044), and the group difference between PTSD + ECT and PTSD only trended towards significance (p = 0.062).

### Sex disaggregated analysis

A RM-ANCOVA between PTSD males (n = 8) and PTSD females (n = 9) revealed no main effect of sex (F_(1,14)_ = 0.05, p = 0.824). However, sex disaggregated analyses were conducted using RM-ANCOVAs in males only and females only due to previous sex specific findings^23^. A marginal main effect of group was observed in TSPO binding between PTSD males (n = 8) and HC males (n = 11) (F_(1,14)_ = 3.222, p = 0.094) where PTSD males had 21% higher [^18^F]FEPPA V_T_ compared to HC males. The biggest group differences were observed in the PFC (16%; p = 0.088) and the amygdala (53%; p = 0.096). There were no group differences (F_(1,17)_ = 0.166, p = 0.689) between PTSD females (n = 9) and HC females (n = 11). Sensitivity analysis did not change this finding.

### Serum cortisol

**Figure 3:**
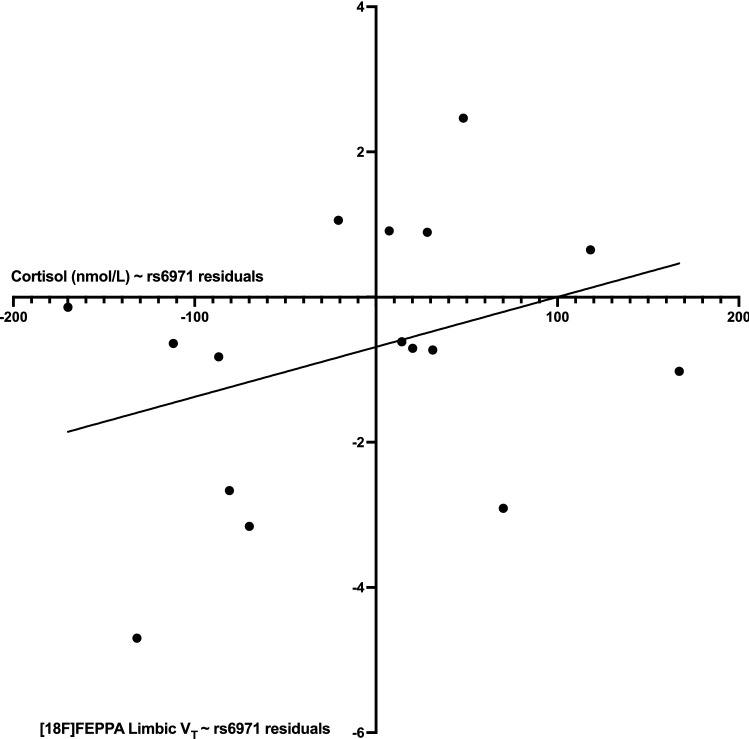
Relationship between cortisol and [^18^F] FEPPA V_T_. Higher serum cortisol concentrations (nmol/L) was correlated (r = 0.530, p = 0.028) with higher [^18^F] FEPPA V_T_ in PTSD participants (n = 16).

Cortisol levels were marginally lower (p = 0.077, % difference: − 21%) in PTSD relative to HC (Supplementary Fig. S2). There was a significant relationship between serum cortisol concentration (nmol/L) and average fronto-limbic [^18^F]FEPPA V_T_ (R = 0.530, p = 0.028) where higher serum cortisol was associated with higher [^18^F]FEPPA V_T_ in PTSD participants (Fig. 3). The relationship between [^18^F]FEPPA V_T_ and serum cortisol was not observed in HC participants (R = − 0.130, p = 0.597).

### Relationships between TSPO binding and symptoms

Correlations between the residuals (regressed onto TSPO genotype) of symptoms, [^18^F]FEPPA V_T_ and hormone concentrations were conducted to investigate potential relationships. There were no relationships between [^18^F]FEPPA V_T_ and clinical symptoms or PTSD severity (p > 0.2).

## Discussion

The current study did not observe a significant group difference in fronto-limbic TSPO binding between HC and PTSD participants, although the PTSD cohort exhibited non-significantly higher (~ 30%) TSPO binding in the amygdala. We did observe greater TSPO binding in PTSD participants who regularly use cannabis, in male participants with PTSD (non-significant), and in those exposed to childhood trauma (non-significant). Additionally, serum cortisol levels were positively related to TSPO binding in fronto-limbic regions within the PTSD group only.

In contrast with the only other in vivo PET/TSPO investigation in people diagnosed with PTSD (14% decrease in TSPO binding)^23^, the current study did not observe lower TSPO binding. Instead, we report nominally higher TSPO binding (6.5–30%) in PTSD relative to HC. These findings are in line with most preclinical studies suggesting exposure to acute and chronic stress leads to microglia activation^34^ but depart from other reports suggesting a downregulation of TSPO in rodents exposed to long-term stress^35–37^, and a recent study by our group (in which 13 participants from the current study were enrolled) that observed a trend for lower MAO-B binding (a marker for astrocytes; another type of glia cell) in PTSD^38^. Our data are also consistent with in vivo findings that TSPO binding is positively related to greater PTSD symptoms in World Trade Centre first responders^28^ and is elevated in MDD^39^, a disorder with overlapping symptomatology.

There are several factors that can account for the difference in findings between the current study and results of Bhatt et al.’s.^23^ PET/TSPO study. First, while our study focused on occupation-related PTSD acquired in adulthood, Bhatt et al.^23^ primarily enrolled participants with complex trauma where traumatic exposure (e.g., sexual violence, assaults) was encountered across the lifespan and during developmental periods (i.e., early life). The type of trauma, frequency (chronic vs sub-chronic) and recency of traumatic events could also have contributed to the divergent findings^17^. For example, preclinical literature indicates that sub-chronic exposure to unpredictable stress increases microglia activation whereas a chronic regimen decreases activation^14^. Alternatively, the difference in findings may be due in part to a higher proportion of cannabis users enrolled in our study which may have increased TSPO binding^40^.

The largest group difference in TSPO binding was observed in the amygdala, where PTSD participants, albeit non-significant, had 30% higher binding compared to HC participants (Cohen’s *d*: 0.46). The amygdala nuclei have a well-established role in fear response and in modulating negative affect and emotion, and amygdala hyperactivity has been robustly implicated in PTSD^41^. Microglia activation in the amygdala in animal models exposed to stress has been reported by some^42–44^ but not all studies^45,46^. Interestingly, studies showing stress-induced deramification of microglia in the amygdala have linked microgliosis in this brain area with an anxiety phenotype^47^, while other studies show inhibition of microglial activity in this region attenuates stress-induced visceral responses^48^. Together, these findings and our preliminary data suggest that microgliosis in the amygdala might be involved in PTSD.

Our finding of higher TSPO binding in PTSD among those who regularly use cannabis is in line with another in vivo investigation reporting 23% higher TSPO binding in frequent cannabis users (~ 8–9 g/week) compared to non-cannabis users^40^. Regular cannabis use can downregulate the CB1 receptor, and this is associated with a pro-inflammatory phenotype in microglia^49^, which may have increased TSPO binding. This is an important mechanism to understand since cannabis is currently being explored as a therapeutic treatment for PTSD. It is important to note that five of six cannabis users within the PTSD group are males and we also reported higher (non-significant) TSPO binding among males with PTSD compared to HC males. Considering preclinical research^50^ investigating sex specific differences in response to stress, this finding could be sex driven. Another possible reason for higher TSPO binding in regular cannabis users is lack of SSRI use in this subgroup. Of the 10 PTSD participants on SSRIs, only one reported regular cannabis use and our sensitivity analysis revealed lower TSPO binding in PTSD participants using SSRIs. Previous studies^51–53^ suggest that duration and/or state SSRI use is associated with lower TSPO V_T_.

### Relationship between cortisol and TSPO binding

We report a positive relationship between serum cortisol and fronto-limbic TSPO binding in PTSD participants. To our knowledge, this is the first study reporting a relationship between peripheral cortisol concentrations and brain TSPO binding in humans with PTSD. This positive relationship is at odds with preclinical research demonstrating a negative relationship between peripheral cortisol concentrations and microglia activation^54^. Furthermore, a recent study^55^ observed increased brain cortisol (measured by PET imaging of a cortisol producing enzyme) in people with PTSD. Interestingly, brain cortisol was negatively related with PTSD symptoms and there was no relationship observed between central and peripheral cortisol levels. It is likely that systemic biological processes may not be reflective of (and in fact, dysregulated from) central processes; for example, in their previous study, Bhatt et al.^23^ reported a negative relationship between TSPO binding and peripheral C-reactive protein (CRP) concentrations in people with PTSD. With no observed relationship with PTSD symptoms, it is difficult to understand the clinical significance of this relationship, especially considering TSPO binding does not differentiate between M1/M2 microglia activation. Nonetheless, that this relationship was observed in the PTSD study group only, further supports a role for microglia and HPA axis involvement in the pathology of PTSD. Considering the effects that GCs, including cortisol, can have on fear memory and memory reconsolidation and extinction^56^, this relationship is important to study further. It is also possible the relationship between cortisol and TSPO binding might be related to the functional role of TSPO in neurosteroid synthesis and steroidogenesis rather than microglia activation. It is also unclear if cannabis use or sex differences are driving this relationship as males with PTSD reported significantly more cannabis use (which could affect cortisol concentrations^57^) and there were no differences in cortisol concentration between males with PTSD compared to HC participants.

### Limitations

The current study focusing on occupation-related PTSD contributes to a growing body of literature investigating the potential role of TSPO/microglia in PTSD but is not without limitations. First, we reported higher TSPO binding among PTSD participants compared to HC in the amygdala. While the fear conditioning role of the amygdala has been well established in PTSD, this finding should be interpreted with caution. The amygdala is a small subcortical region in the brain that is difficult to fit accurately. Region specific TSPO group differences in the amygdala should be investigated in larger sample sizes in future studies. We also investigated TSPO binding in a heterogenous sample (cannabis use, medication use). While this is reflective of the generalized occupation-related PTSD cohort, we were underpowered to fully explore the effect of independent factors on TSPO binding. Additionally, it would have been informative to characterize trauma exposure in our healthy control cohort. Finally, we employed a TSPO PET ligand as an index of microglia cells, however TSPO is also present on other cells within the brain (astrocytes, endothelial cells) and is not solely specific to microglia cells.

### Conclusions and next steps

Although we report no significant difference in TSPO binding between HC and PTSD groups, we observed a higher TSPO binding in PTSD participants who report regular cannabis use, PTSD participants who report a history of exposure to childhood trauma, and a trend for higher TSPO binding among male participants with PTSD, which merits further investigation in a larger sample size. Despite earlier findings of lower TSPO binding in PTSD^23^, our findings suggest microgliosis cannot be ruled out. Factors such as cannabis exposure, biological sex, and lifespan exposure to trauma contribute variability to the finding and need to be cautiously considered.

## Methods

### Participants

Twenty participants with occupation-related PTSD and twenty-three HC provided written informed consent and completed a [^18^F]FEPPA PET scan and one magnetic resonance imaging (MRI) scan as part of a study approved by the Centre for Addiction and Mental Health Research Ethics Board. All methods were performed in accordance with the relevant guidelines and regulations. Participants with PTSD were recruited through the St. Joseph’s Operational Stress Injury (OSI) clinic, CAMH affiliated clinics, and from the community. Healthy research participants were recruited from the Greater Toronto Area (GTA) and surrounding regions, using advertisements via flyers and web postings.

Participants completed a comprehensive medical and psychiatric screening (Semi‐Structured Clinical Interview for Diagnostic and Statistical Manual of Mental Disorders (DSM)-IV/5^58^, and several questionnaires characterizing mood^31–33^, nicotine dependence^59^, and traumatic experiences^60^. Saliva samples (Oragene DNA, DNA Genotek Inc., Ottawa, Canada) were obtained to genotype for the TSPO rs6971 polymorphism which affects [^18^F]FEPPA binding^61^. Participants were eligible to participate if they were 17 years old or older, physically healthy with no current or previous DSM Axis I diagnosis except co-morbid mood disorder with (PTSD group only), and were not low affinity binder (LAB)s for TSPO polymorphism. PTSD participants were included if they met criteria for current PTSD diagnosis. PTSD symptoms were assessed via the PCL^62^ and PTSD Symptom Scale^63^.

### PET and MRI image acquisition and image analysis

All PET and MRI scans were performed at the Brain Health Imaging Centre at CAMH. On scan day, urine toxicology and pregnancy (in female participants) were assessed, and breath alcohol (breathalyzer) and expired carbon monoxide (CO) were measured to rule out recent use of alcohol and smoking. Participants were instructed not to use any medications with anti-inflammatory properties, or drugs of abuse for a minimum of 1 week before the scan.

[^18^F]FEPPA synthesis (produced by reaction of cyclotron produced [^18^F]-fluoride with a tosylate precursor) has been described in detail^64^. The PET scanning was done as previously reported^65^. Briefly, a brief transmission scan was first acquired to correct PET images for attenuation using a single photon point source [CS-137]. This was followed by a 120-min emission scan after an intravenous saline solution of 5.1 ± 0.26 mCi of [^18^F]FEPPA administered at approximately a 1-min bolus into the antecubital vein followed by approximately 10 ml of saline. PET images were corrected for attenuation, reconstructed by filtered back projection algorithm using an HANN filter at Nyquist cutoff frequency, and reconstructed into a series of 33 time frames (one frame of variable length, followed by frames comprising 5 × 30 s, 1 × 45 s, 2 × 60 s, 1 × 90 s, 1 × 120 s, 1 × 210 s, and 22 × 300 s).

Arterial blood samples were taken continuously for the first 22.5 min at a rate of 2.5 ml/min using an automatic blood sampling system (ABSS, Model #PBS-101 from Veenstra Instruments, Netherland). In addition, 7-ml manual samples were drawn at 2.5, 7, 12, 15, 20, 30, 45, 60, 90, and 120 min to determine the metabolization of the radioligand. Input functions for the kinetic analysis were generated as previously described^65^.

PET images were analyzed using a region-based method in which radioactivity was extracted from automatically delineated MRI-based whole brain anatomical regions of interest (ROIs) as described in Ref.^66^. Time activity curves (TAC) extracted in 6 ROIs of the fronto-limbic neurocircuitry including the hippocampus, amygdala, prefrontal cortex (PFC), striatum, insula, and anterior cingulate cortex (ACC) and in arterial plasma were fitted using a two-tissue compartment model (2-TCM) (as described in Ref.^67^). [^18^F]FEPPA distribution volume (V_T_), the ratio at equilibrium of the radioligand concentration in tissue to that in plasma, was measured in each of the 6 ROIs and used as the main TSPO binding parameter.

### Serum cortisol analysis

Peripheral blood samples were drawn on PET scan day prior to tracer injection between 12 and 3 p.m. (except for one HC sample at 9 a.m. and one PTSD sample at 11 a.m.). Briefly, blood samples were drawn into 4 mL serum vacutainers and left to equilibrate at room temperature for 1 h prior to centrifugation for 20 min at room temperature. The supernatant was aliquoted and stored at − 80 °C until analysis. Serum cortisol concentrations were analyzed by solid-phase chemiluminescent immunoassay via the Immulite 1000 analyzer (Siemens Healthineers Global, Erlangen, Germany).

### Statistical analysis

Descriptive statistics were calculated for participant demographics and clinical information. Group differences were evaluated by independent samples T-tests, Mann–Whitney U tests, or Chi square tests where appropriate. [^18^F]FEPPA V_T_ data was assessed for normality (Shapiro–Wilk Test) and data was assessed for outliers (Z score > 3) and influential data points (Cook’s Distance > 0.15); sensitivity analysis were performed where appropriate. A repeated measures analysis of covariance (RM-ANCOVA), controlling for TSPO genotype with ROI (amygdala, hippocampus, prefrontal cortex (PFC), striatum, insula, and anterior cingulate cortex (ACC)^23,38^) as a repeated measure was used to estimate group differences in [^18^F]FEPPA V_T_ (Group [2] × ROIs [6]). Demographic variables (e.g., age and sex) were entered into the model. Sphericity was assessed with the Mauchly's Test of Sphericity and corrected using Greenhouse–Geisser where necessary. Post-hoc pairwise comparisons were used to dissect the interaction. [^18^F]FEPPA V_T_ values were regressed onto TSPO genotype and two-tailed Pearson correlations were employed to evaluate possible correlations between [^18^F]FEPPA V_T_ residuals and duration of PTSD, serum cortisol concentrations, and symptom severity. All statistical analysis was conducted using IBM SPSS Statistics 27 (Armonk, New York, USA).

## Supplementary Information

Supplementary Information.

## Data Availability

The data that support the findings of this study are available upon request from the corresponding author (IB).
