# Supplementary material for: Investigating TSPO levels in occupation-related posttraumatic stress disorder
Source: Sci Rep. 2023 Mar 27;13:4970. doi: 10.1038/s41598-023-31327-y (PMC10041517; doi:10.1038/s41598-023-31327-y)
Supplement: Supplementary file 1 — Supplementary Information. [file 41598_2023_31327_MOESM1_ESM.docx]

**SUPPLEMENTARY MATERIAL**

*Whole Brain Analysis*

A RM-ANCOVA controlling for TSPO genotype and age was conducted across 10 ROIs: amygdala, hippocampus, insula, temporal lobe, PFC, striatum, thalamus, ACC, occipital lobe, and cerebellum. Whole brain analysis revealed no main effect of group (F_(1,35)_=1.007, p=0.323) and a marginal ROI*Group interaction (F_(4.053, 141.868)_=1.535, p=0.194). PTSD participants had 14% higher TSPO binding compared to HC. Sensitivity analysis of the whole brain revealed similar results: no main effect of group (F_(1,35)_=0.660, p=0.422) (PTSD participants had 9% higher TSPO binding compared to HC) and a marginal ROI*Group interaction (F_(4.053, 141.868)_=2.140, p=0.091), where the largest group difference was observed in the amygdala (p=0.114).

| Table S1: Whole Brain Analysis | | | | |
| --- | --- | --- | --- | --- |
| ROI | Group | [^18^F]FEPPA V_T_ | % Difference | p value |
| Amygdala | PTSD | 9.84 | 30% | 0.107 |
|  | HC | 7.52 |  |  |
| Hippocampus | PTSD | 10.19 | 12% | 0.279 |
|  | HC | 8.87 |  |  |
| Insula | PTSD | 10.75 | 6.5% | 0.596 |
|  | HC | 10.08 |  |  |
| Temporal Lobe | PTSD | 11.09 | 11.7% | 0.363 |
|  | HC | 9.93 |  |  |
| Striatum | PTSD | 9.66 | 22% | 0.328 |
|  | HC | 8.5 |  |  |
| Thalamus | PTSD | 12.84 | 13.2% | 0.322 |
|  | HC | 11.34 |  |  |
| ACC | PTSD | 10.47 | 15% | 0.356 |
|  | HC | 9.22 |  |  |
| Occipital Lobe | PTSD | 11.12 | 8.1% | 0.488 |
|  | HC | 10.29 |  |  |
| PFC | PTSD | 11.13 | 16% | 0.406 |
|  | HC | 10.03 |  |  |
| Cerebellum | PTSD | 11.22 | 11.5% | 0.357 |
|  | HC | 10.06 |  |  |
|  |  | | | |

| Table S2: Participant Demographics: PTSD regular cannabis users vs. non-users | | | |
| --- | --- | --- | --- |
|  | PTSD Non-users  n=12 | PTSD Cannabis Users  n=6 | p value |
| Age, years | 47±10.8 | 40±10.3 | 0.166 |
| Sex, male n(%) | 3 (25) | 5 (83) | 0.027 |
| NIH race, caucasian n (%) | 8 (66) | 6 (83) | 0.317 |
| BMI | 27.4±3.3 | 24.77±3.5 | 0.203 |
| TSPO genotype, HAB (%) | 6 (50) | 3 (50) | 0.858 |
| Years of education | 15.9±2.9 | 15±1.7 | 0.543 |
| Cigarette Smokers n (%) | 3 (25) | 0 (0) | 0.058 |
| Positive THC on PET day n (%) | 7 (38) | 4(23) | 0.123 |
| Alcohol drinks / week | 2.71±2.4 | 2.69±1.9 | 0.959 |
| SSRI Users n(%) | 9 (75) | 1 (17) | 0.009 |
| Co-morbid MDD n(%) | 3 (25) | 2 (33) | 0.793 |
| History of TBI n(%) | 6 (50) | 2 (33) | 0.402 |
| *Questionnaires* |  |  |  |
| BDI, mean ± SD | 24±13 | 20±10 | 0.580 |
| GAD-7, mean ± SD | 10.45±6.4 | 7.75±3.6 | 0.450 |
| PHQ-9, mean ±S D | 14.8±8.4 | 10±5.7 | 0.379 |
| PCL, mean ± SD | 57.9±18.8 | 48.3±16.1 | 0.304 |
| PSS, mean ± SD | 32.6±16.2 | 25.4±10.3 | 0.387 |
| Values are mean +/- SD unless otherwise indicated  Body Mass Index (BMI); High Affinity Binder (HAB); Beck Depression Inventory (BDI); PTSD Symptom Scale (PSS); PTSD Checklist (PCL). | | | |


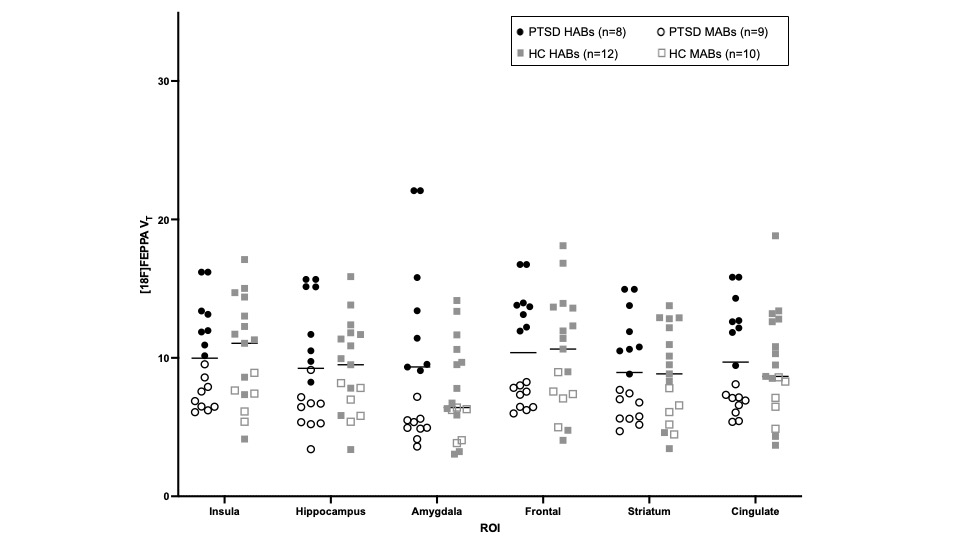


**Figure S1:** Sensitivity Analysis [18F]FEPPA V_T_ scatterplot**.** Capping the influential data point at the second highest value revealed no main effect of group (F_(1,35)_=0.472, p=0.496) and a significant ROI*Group interaction (F_(2.563, 89.704_=3.957, p=0.015). PTSD participants had a 29% higher TSPO binding compared to HC in the amygdala (p=0.114).

**Figure S2:** Scatter plot of cortisol concentrations between PTSD (n=18) and HC (n=20). Serum cortisol was marginally lower (p=0.077, % difference: -21%) in PTSD participants compared to HC participants.
